# Sown alfalfa pasture decreases grazing intensity while increasing soil carbon: Experimental observations and DNDC model predictions

**DOI:** 10.3389/fpls.2022.1019966

**Published:** 2022-11-21

**Authors:** Lijun Xu, Liming Ye, Yingying Nie, Guixia Yang, Xiaoping Xin, Bo Yuan, Xiufang Yang

**Affiliations:** ^1^ Institute of Agricultural Resources and Regional Planning, Chinese Academy of Agricultural Sciences, Beijing, China; ^2^ Department of Geology, Ghent University, Ghent, Belgium; ^3^ Chifeng Institute of Agricultural and Animal Sciences, Chifeng, China

**Keywords:** alfalfa, *Medicago sativa* L., grazing intensity, stocking rate, soil organic carbon, forage yield, calibration, climate change

## Abstract

**Introduction:**

Grasslands are the most important land use in China and have experienced extensive degradation in the past few decades due to overgrazing. However, regionally viable solutions to grazing intensity alleviation remained elusive to date.

**Methods:**

Here, we evaluated the grazing intensity effects of sown alfalfa pastures in northern China using an experiment-modeling combined approach that involved six sites in field experiments and five provinces in DNDC modeling of sown alfalfa pasture’s forage production and carbon sequestration potentials in marginal lands.

**Results:**

Our results showed that the sown alfalfa pasture’s dry-matter yield varied between 4.5 and 9.0 Mg ha-1 under rainfed and irrigated conditions, respectively, from 2025 to 2035. If half of the available marginal lands were mobilized for alfalfa forage production, these yield levels meant that livestock grazing intensity on natural grasslands may drop 8-13% under rainfed and 20-33% under irrigated conditions. Our results also showed that marginal land’s soil organic carbon contents were systematically higher under sown alfalfa pasture than under fallow management by a big margin of 8.5 and 9.9 g kg-1 (i.e., +79 and +95%), under rainfed and irrigated conditions, respectively, during 2025-2035.

**Discussion:**

Overall, these results demonstrated that sown alfalfa pasture on marginal lands represents an effective grassland conservation pathway over the short- to medium-term time horizon based on current technologies.

## 1 Introduction

Climate change is a pressing issue facing humanity on Earth. Although the direct cause of it is the rapid increase in greenhouse gas concentrations in the atmosphere resulting from the large-scale use of fossil fuels since the Industrial Revolution (IPCC, 2019), the role that agriculture and food production have played is by no means negligible. Recent research revealed that greenhouse gas emissions from the food and agriculture sector accounted for 20-30% of the total annual emissions of the world (Smith et al., 2014). Being more environmental-friendly while ensuring food security for more than 10 billion people is the inevitable challenge that world agriculture has to face in the twenty-first century (Ye et al., 2016).

As the most populous country in the world, China is not only the largest producer of staple grains but also the largest producer of livestock. As part of the Eurasian Steppe, grasslands in China possess an area of 3.93 million km^2^, accounting for 12.5% of the total area of the world’s grasslands and 41.4% of China’s total land mass. In 2020, China produced 77.6 million tons of meat, leading the second largest producer, the United States, by a big margin of 50% (NBSC, 2021). Continuous production at this scale has caused substantial changes to the grassland ecosystem in China (Xu et al., 2020). Due to the increase in livestock quantity and changed land use from semi-nomadic systems to a sedentary system, the size and productivity of typical steppes in northern China have markedly decreased (Schröder et al., 2014). In response, forage production in combination with livestock rearing in captivity instead of grazing has been put forward and subsequently recognized as one of the practical countermeasures at regional scales (Robinson et al., 2017). Due to its wide suitability across climatic zones and its overwintering ability to withstand harsh winter conditions in northern China (Xu et al., 2021), alfalfa (*Medicago sativa* L.) is considered one of the best candidate plant species to be used in large-scale forage production in grassland ecosystems and land rehabilitation in agropastoral ecotones (Nasar et al., 2020; Xu et al., 2022).

Alfalfa is a perennial legume that is widely cultivated around the world and often used as high-quality forage. Alfalfa cultivation in China can be dated back more than 2000 years. In 2016, the area of sown alfalfa pastures reached 3.84 million hectares, taking a 40% share of the total acreage of all high-quality forage species (National Animal Husbandry Station, 2017). However, most of the sown acreage occurred in provinces south of the Yellow River. Although its hardiness to withstand the northern winter has recently been proven by field experiments (Xu et al., 2021), alfalfa’s adoption in these provinces is still in the early stage. Integration of alfalfa into the livestock and/or agropastoral systems in northern China has multiple benefits. Alfalfa-enabled crop- and grassland production systems have the potential to improve the land productive capacity by biological nitrogen fixation (Sun and Li, 2019) and by other mechanisms, e.g., plant-plant, plant-soil, and plant-microbe interactions (Malhi et al., 2002; Guo et al., 2019; Wang et al., 2022). In a broader spatial context, mosaic grassland landscapes involving alfalfa have the potential to provide a range of ecosystem services such as water quality preservation, biodiversity protection, biotic regulation, and stability (Asbjornsen et al., 2014; Humphries et al., 2021; Xia et al., 2022). Moreover, from an operational viewpoint, perennial forage production systems can support livestock rearing on the scale of economy, which in turn can displace a substantial part of the market pressure on the grazing grassland and thus is regarded as an effective conservation measure (Robinson et al., 2017). Nevertheless, the forage production should not compete with grassland or cropland for acreage. The local wasteland, abandoned land, and other types of marginal lands should be utilized for this purpose (Chen et al., 2017), as previously demonstrated by the case of Hungary in the 1990s (Valkó et al., 2017).

Therefore, we hypothesize that sown alfalfa pastures on marginal lands in northern China is an effective conservation measure at least at the regional scale in lowering grassland’s grazing intensity *via* increased livestock rearing in captivity. We also hypothesize that regional alfalfa pastures provide a real opportunity for land quality improvement by raising soil organic carbon (SOC) contents. To test our hypotheses at the regional scale, we conducted field experiments of sown alfalfa pastures at six research sites along a geographical transect over a distance of more than one thousand kilometers in northern China. We collected yield and soil data during 2012-2017. To further illustrate the validity of our hypotheses for the near-term future, we simulated alfalfa yield and SOC contents in five northern provinces in China for the period 2025-2035 using the DNDC model, and quantitatively analyzed the alfalfa-based forage system’s potential in lowering the grazing intensity on grasslands and sequestering carbon (C) in marginal lands across northern China.

## 2 Materials and methods

### 2.1 Study sites

**Figure 1:**
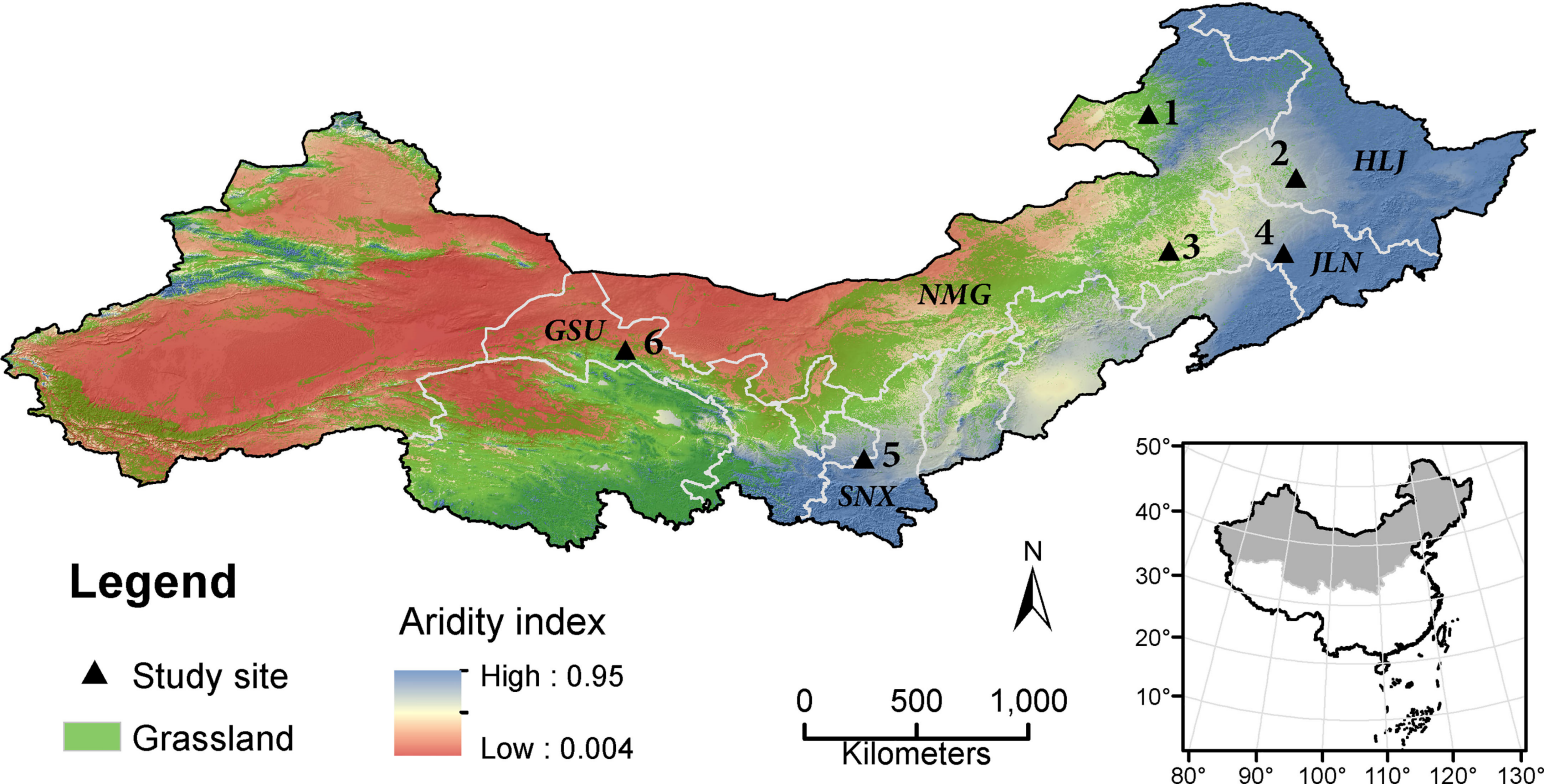
The study sites in relation to the regional distribution of grasslands and climatic aridity. Grassland is extracted from ESA CCI land cover v2.0.7 for the year 2015. The aridity index is evaluated as the rainfall to potential evapotranspiration ratio, averaged for the period 1970-2000. Site 1, Hulunber; 2, Suihua; 3, Chifeng; 4, Gongzhuling; 5, Yulin; and 6, Jiuquan. Provinces: HMG, Inner Mongolia; HLJ, Heilongjiang; JLN, Jilin; SNX, Shaanxi; GSU, Gansu.

The field experiment was conducted in six sites in northern China, including Hulunber, Suihua, Chifeng, Gongzhuling, Yulin, and Jiuquan (**Figure 1**). The climatic and soil characteristics of these sites are given in **Table 1**.

**Table 1:** Climatic and soil characteristics of the research sites.

Site	Province	MAT (°C)	FFP (d)	MAP (kg m^-2^)	PET (kg m^-2^)	Aridity index	Soil unit (WRB, 2015)	Bulk density (Mg m^-3^)
Hulunber	Inner Mongolia	-2.4	110	295	984	0.30	Luvic Kastanozems	1.29
Suihua	Heilongjiang	2.9	130	470	1140	0.41	Gleyic Phaeozems	1.37
Chifeng	Inner Mongolia	5.5	130	350	1480	0.24	Haplic Kastanozems	1.41
Gongzhuling	Jilin	4.5	144	595	1172	0.51	Haplic Phaeozems	1.24
Yulin	Shaanxi	9.7	180	397	1208	0.33	Calcaric Cambisols	1.24
Jiuquan	Gansu	7.5	135	87	1639	0.05	Luvic Gypsisols	1.36

MAT, mean annual temperature; FFP, frost-free period; MAP, mean annual precipitation; PET, potential evapotranspiration.

### 2.2 Field experiments

Field trials were conducted at each of the six experimental sites for five consecutive years during 2012-2017 to test the suitability and to measure the aboveground dry-matter yield of alfalfa. Field management was kept consistent across experimental sites. At each site, four parallel trial blocks of 1 m × 3 m were used to represent four replicates of alfalfa pastures; blocks were separated by a space of 0.5 m in width. Seeds were sown in 0.015-0.025 m soil depth and rows with a 0.2 m inter-row spacing. Seed density was kept at 18 kg ha^-1^ for all sites. Diammonium phosphate was applied as the base fertilizer at a rate of 150 kg ha^-1^. Alfalfa plants were harvested 3-4 times a year, as stipulated by the management calendar (**Table 2**).

**Table 2:** The alfalfa management calendar used in the field experiments.

Site	Cultivar	Land use	Sowing (2012)	Intermediate Cutting			Annual harvest
				First	Second	Third	
Hulunber	Gongnong-1	Abandoned land	25 Jun	17 Jul	20 Jul	None	25 Aug
Suihua	Gongnong-1	Fallow land	4 May	15 Jun	29 Jul	15 Aug	30 Aug
Chifeng	Aohan	Fallow land	15 Apr	27 May	20 Jun	25 Jul	10 Sep
Gongzhuling	Gongnong-1	Abandoned land	20 Apr	8 Jun	20 Jul	None	20 Sep
Yulin	Aergangjin	Fallow land	11 Apr	8 Jun	20 Jul	15 Aug	30 Aug
Jiuquan	Aergangjin	Fallow land	10 May	22 May	30 Jun	28 Jul	5 Aug

A quadrat of 0.5 m × 0.5 m was used at each cutting time and at harvest to determine biomass yield. The quadrat was laid down at three random points within each trial block. Alfalfa plants inside the quadrat were manually cut at 0.05 m above the soil surface. The plants were taken to the laboratory and oven-dried at 65°C for 24 hours or until a constant weight was reached. The dried plants were weighed to obtain the dry weight.

Three soil samples of 0-0.3 m depth were randomly taken using a soil drill (0.07 m inner diameter; 0.1 m height, New Landmark Instrument Co., Ltd., Beijing) near the quadrat. Samples from the same sampling point were mixed to form a composite sample. All samples were air-dried in the laboratory. SOC was determined using the potassium dichromate oxidation method (Patrick et al., 1996).

Soil gas fluxes were measured using the opaque static chamber method (Zhang et al., 2010). The static chamber system consisted of a stainless-steel frame (open top and bottom, 0.5 m in length × 0.5 m in width × 0.1 m in height) that was driven into the soil and a stainless-steel chamber (open bottom, 0.5 m L × 0.5 m W × 0.5 m H) that was placed tightly in the base groove during the sampling period. The frame was inserted into the soil to a depth of 0.1 m below the soil surface. A cover was placed on top during sampling times and removed afterward. A fan of 0.1 m in diameter was installed at the top of each chamber to generate turbulence when the chamber was closed. The external surface of each chamber was covered with white plastic foam to minimize the effects of direct radiative heating during sampling. Three replicate chambers were randomly established in each plot and used for simultaneous measurements. The headspace in each chamber was sampled at intervals of 0, 10, 20, and 30 minutes after the chamber was closed. The gas was transferred immediately into a pre-evacuated 0.05 litter airbag using a 0.06 litter plastic syringe (Hede Inc., Dalian, China). This sampling procedure was executed every 3 days during the growing season (June-October) from 2013 to 2015 in Hulunber. All measurements were taken between 9 and 11 a.m. The CH_4_, N_2_O, and CO_2_ concentrations of the gas samples were analyzed in the laboratory using gas chromatography (Agilent 7890 A, Agilent Technologies Limited Co., USA).

Soil temperature and soil moisture were measured *in situ* in 0-0.1 m soil depth. Soil temperature was measured using a TJ2 soil temperature recorder (Beijing Hezhong Bopu Technology Development Co., Ltd, China). Soil moisture was measured using an HS2 portable soil moisture sensor (Decagon Devices, USA).

### 2.3 DNDC modeling

The DNDC model (Li et al., 2003) was employed to simulate alfalfa yield in five northern provinces in China for the period between 2025 and 2035. These provinces included Inner Mongolia, Heilongjiang, Jilin, Shaanxi, and Gansu (**Figure 1**). The alfalfa pastures were assumed on marginal lands only. The marginal land SOC contents with and without alfalfa pastures were also simulated.

The DNDC (DeNitrification DeComposition) model was first described by Li et al. (1992) as a biogeochemical simulation model for predicting N_2_O, CO_2_, and N_2_ emissions from agricultural soils in the U.S. After many years of development, DNDC can now be used as a plot-scale, process-based model driven by climate, soil and crop parameters to simulate crop growth, greenhouse gas emissions, SOC and other soil properties not only in the crop but also in livestock and grassland systems (Levy et al., 2007; Gilhespy et al., 2014; Xu et al., 2018; Khalil et al., 2020). To address the unique characteristics of intensive cropping systems in China, Li et al. (2017) included typical Chinese cropping systems (e.g., double and triple paddy rice) into the DNDC model. In addition, the support for common Chinese cultivars of alfalfa was also included.

The DNDC consists of two modules. The first module simulates crop growth and C and N cycling in soil by using three sub-models of soil climate, crop growth, and decomposition. The second module simulates biogeochemical processes related to soil environmental factors by using sub-models of nitrification, denitrification, and fermentation. Crop growth is simulated based on daily accumulations of photosynthesis, respiration, C allocation, water and N uptake by crops (He et al., 2019). Both water and N uptake rely on several factors such as soil N distribution, soil moisture content, and root length, etc. Water utilization depends on potential transpiration linked with leaf area index and climatic conditions. Water stress is simulated when potential transpiration is relatively higher than normal or actual water supply (Zhang et al., 2017). The C pools are divided into four SOC pools, namely, plant residue (litter), microbial biomass, active humus, and passive humus. The litter pool was further divided into three sub-pools of very labile, labile, and resistant litter based on their different C/N ratios and decomposition rates. The DNDC model predicts SOC dynamics mainly by calculating C input (litter, crop residues, root exudates, or manure application) and C output (SOC decomposition or erosion). Litter incorporated into the soil is broken down by soil microbes, partly served energy of the microbes, and partly turns into microbial biomass. After the death of microbes, the microbial remains turn into an active humus pool by decomposition. Then, active humus can be further utilized by microbes to turn into a passive humus pool (Li et al., 2016).

A three-step procedure was followed in this study to simulate alfalfa yield and SOC: (1) the DNDC model was calibrated to each experimental site by adopting a set of finetuned biophysical parameters for alfalfa (**Table S1**); (2) the model was validated using site-specific measurements of soil temperature, moisture, soil gas flux, alfalfa yield, and SOC content; and (3) regional forage production and C sequestration potentials of sown alfalfa pastures on marginal lands in the five northern provinces under three contrasting IPCC SSP climate scenarios between 2025 ad 2035 were simulated.

### 2.4 Model evaluation

The DNDC model’s performance in predicting alfalfa yield and SOC was evaluated using the statistical metrics of mean absolute error (MAE), mean absolute percentage error (MAPE) and root mean square error (RMSE), which were obtained using Equations 1, 2 and 3, respectively:


(1) $$MAE=\sum_{i}\left|{\hat{y}}_{i}-y_{i}\right|/N$$

(2) $$MAPE=\sum_{i}\left(\left|{\hat{y}}_{i}-y_{i}\right|/y_{i}\cdot 100\right)/N$$

(3) $$RMSE=\sqrt{\sum_{i}{\left({\hat{y}}_{i}-y_{i}\right)}^{2}/N}$$

where *y* is the field observed value; $\hat{y}\;$ is the model-predicted value and *N* is the sample size; additionally, the mean absolute percentage accuracy (MAPA) was referred to as the overall prediction accuracy, which was derived from *MAPA*=100−*MAPE* .

### 2.5 Climate scenarios

**Figure 2:**
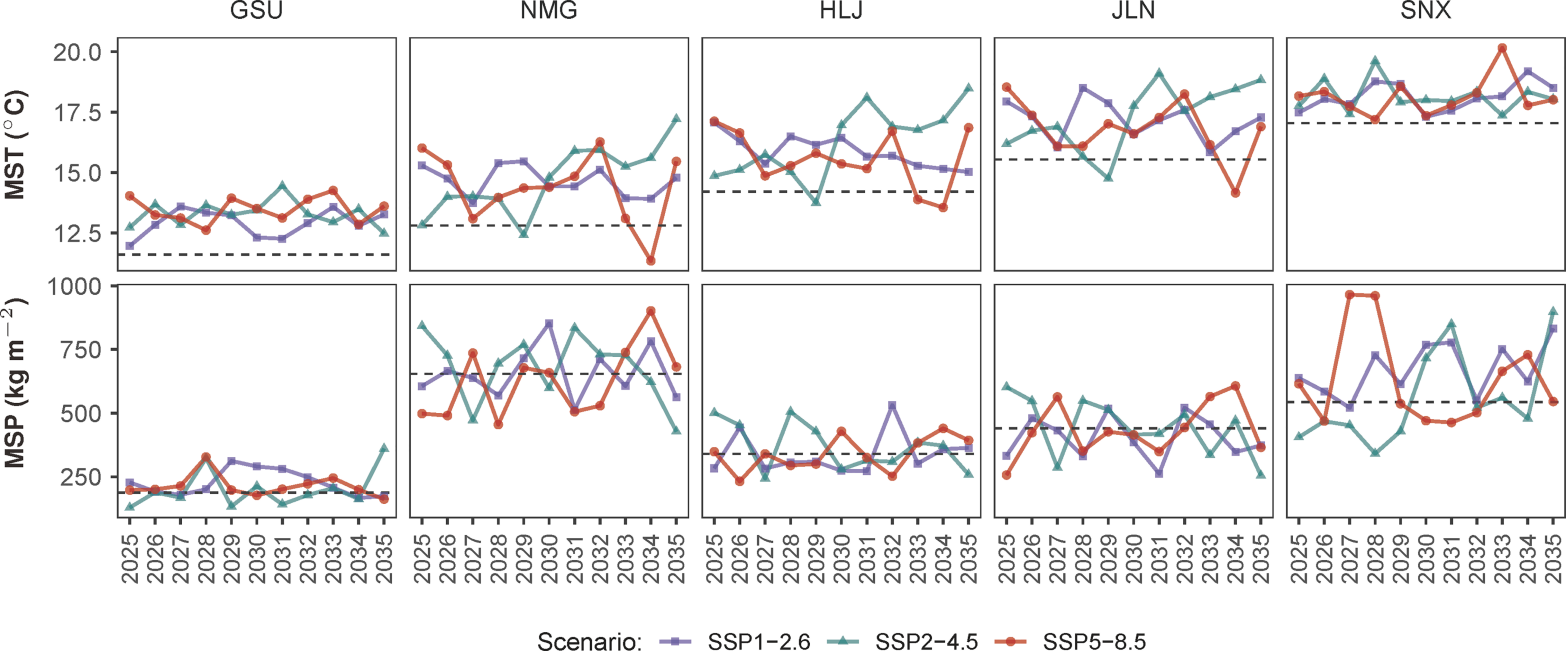
Climatic variations during the alfalfa growing seasons from 2025 to 2035 in six northern provinces in China based on the BCC-CSM2 global circulation model simulations driven by the SSP1-2.6, SSP2-4.5, and SSP5-8.5 scenarios. MST, mean seasonal temperature; MSP, mean seasonal precipitation. The dashed horizontal lines represent the province-specific 20^th^-century mean.

Future climatic data were generated by the Beijing Climate Center’s Climate System Model 2 Medium Resolution version (BCC-CSM2-MR) (Wu et al., 2019) coupled with three contrasting Shared Socioeconomic Pathway (SSP) scenarios, namely, SSP1-2.6, SSP2-4.5, and SSP5-8.5 (Meinshausen et al., 2020). The BCC-CSM2-MR was a participating model in the Coupled Model Intercomparison Project Phase 6 (CMIP6) that supported the IPCC’s Sixth Assessment Report (Eyring et al., 2016). The model’s simulation results were provided at a grid resolution of 1.125 degrees or roughly 100 km in northern China. Descriptions of the three abovementioned SSPs are given in **Table 3**. The BCC-CSM2-MR model-simulated variations in mean temperature and precipitation during the annual growing seasons of alfalfa (MST and MSP, respectively) in the five northern provinces from 2025 to 2035 are presented in **Figure 2**.

**Table 3:** Overview of the climate change scenarios used in this analysis.

Scenario	SSP1-2.6	SSP2-4.5	SSP5-8.5
Alias	“Next-best” scenario	“Middle-of-the-road” scenario	“Business-as-usual” scenario
Characterization	Severe emission cuttings; climate policy; sustainable development	Socioeconomic trend toward sustainability but slow	Fast-growing economies; energy-intensive lifestyles
Radiative forcing by 2100	2.6 W m^-2^	4.5 W m^-2^	8.5 W m^-2^
CO2 concentration by 2100	445 ppm	600 ppm	1135 ppm
Warming by 2100	1.8°C	2.7°C	4.4°C

## 3 Results

### 3.1 Model calibration and validation

#### 3.1.1 Soil temperature, moisture, and greenhouse gas fluxes

**Figure 3:**
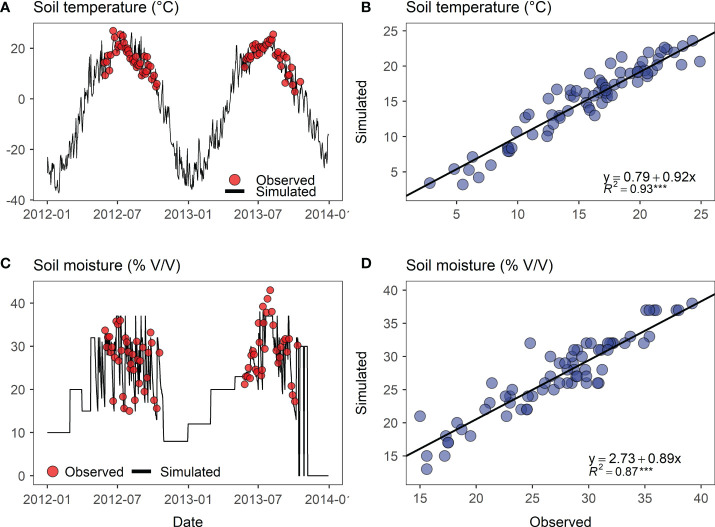
Relationship between the simulated versus observed soil temperature (0-10 cm) and soil moisture (0-10 cm) of the sown alfalfa pasture field at the Hulunber site. **(A, C)** Field observed soil temperature and moisture (red dots) superposed with DNDC-simulations (black curves); **(B, D)** Scatter plots of observed versus simulated soil temperature and moisture. The solid black line represents the linear trend fitted to the simulated versus the observed data. ***p < 0.001.

**Figure 4:**
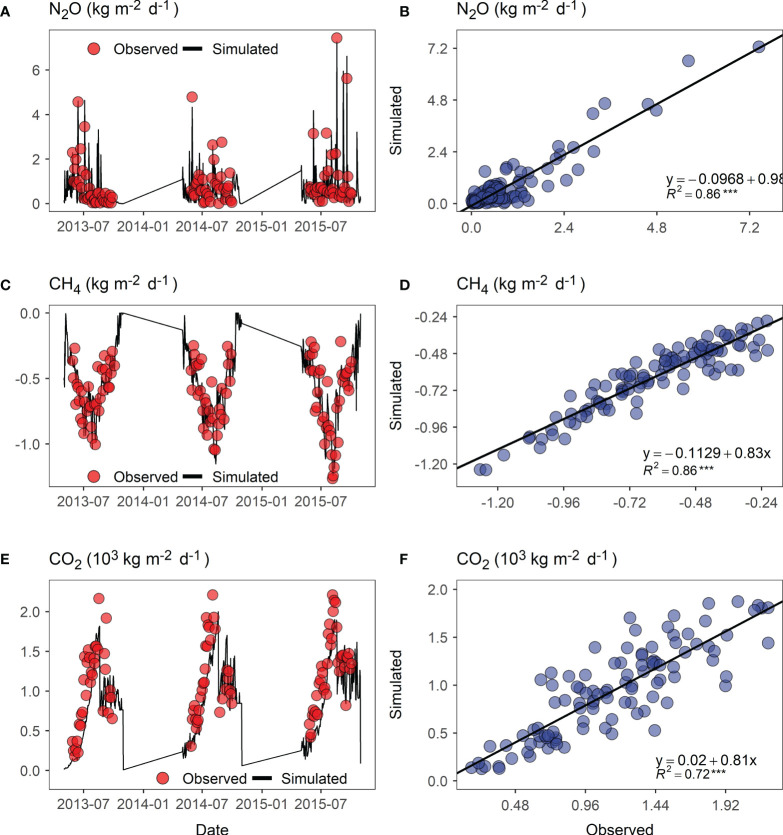
Relationship between the simulated versus observed soil greenhouse gas fluxes of the sown alfalfa pasture field at the Hulunber site. Positive flux represents gas emission from the soil, whereas negative flux represents gas uptake by the soil. **(A, C, E)** Field observed N_2_O, CH_4_, and CO_2_ fluxes (red dots) superposed with DNDC-simulations (black curves); **(B, D, F)** Scatter plots of observed versus simulated soil N_2_O, CH_4_, and CO_2_ fluxes. The solid black line represents the linear trend fitted to the simulated versus the observed data. ***p < 0.001.

To examine whether the DNDC model was adequately calibrated to the biophysical processes on a per-experimental site basis, we plotted the model simulation results against the daily in-situ measurements from the Hulunber site as an example (**Figures 3** and **4**). We used two growing seasons for soil temperature and moisture and three seasons for soil N_2_O, CH_4_, and CO_2_ fluxes. The monitoring results showed that variations in soil temperature, moisture, and gas fluxes followed a typical single-peak pattern per growing season. However, in the first year of the experiment, no clear pattern was observed for soil moisture, showing that alfalfa plants were in an early development stage and their dominance in vegetation evapotranspiration had not been established. A comparison between the simulated and the monitored data revealed that the temporal variations and patterns in either soil temperature, soil moisture, or soil greenhouse gas fluxes were closely reproduced by the DNDC model. The obtained linear trends between the model simulations and the in-situ measurements showed that the slope coefficients were close to 1, whereas the intercept coefficients were close to 0. The coefficients of determination (R^2^) were evaluated to be 0.93 for soil temperature, 0.87 for soil moisture, 0.86 for soil N_2_O and CH_4_ fluxes, and 0.72 for soil CO2 flux.

#### 3.1.2 SOC and alfalfa yield

**Figure 5:**
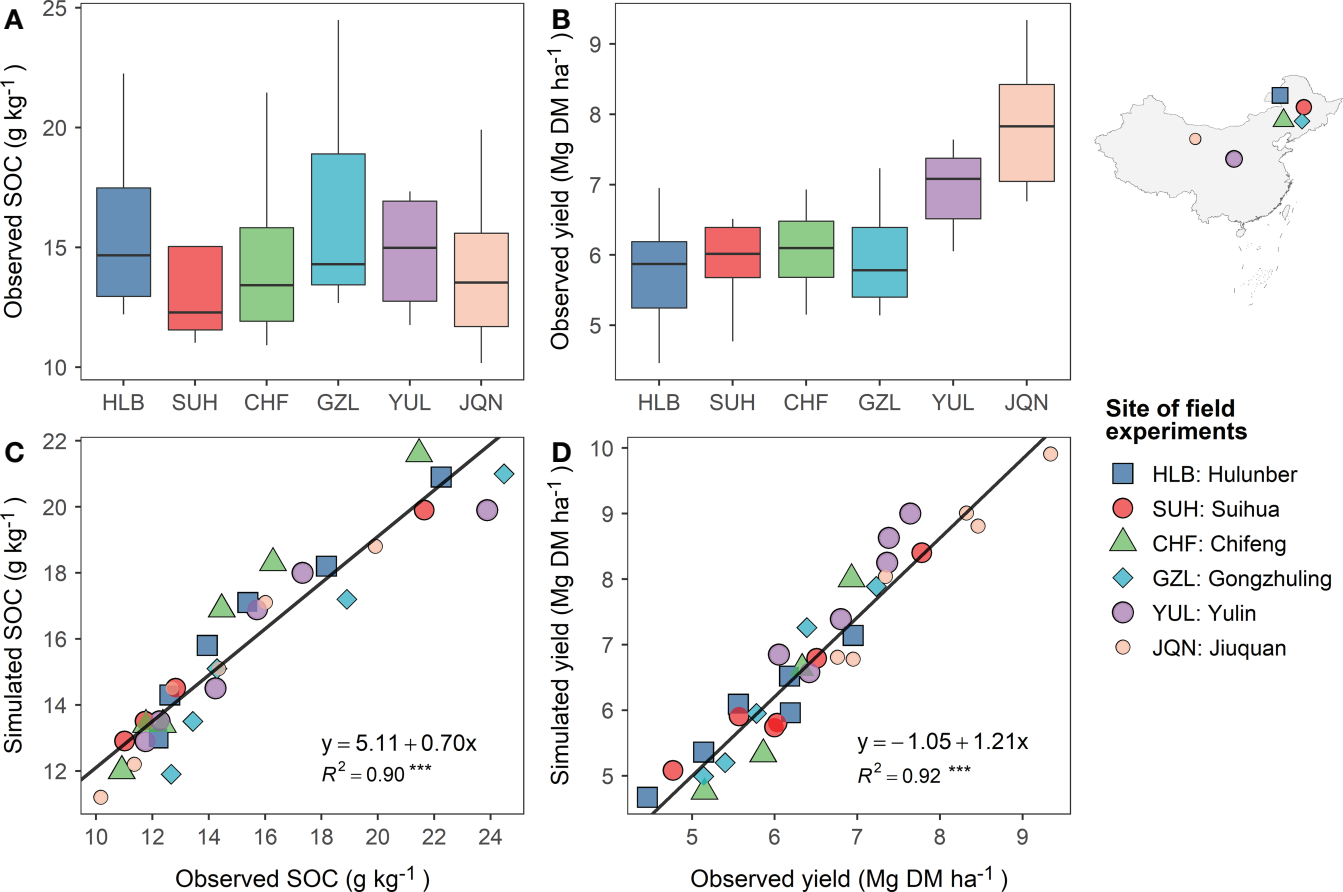
Field observed 0-30 cm soil organic carbon (SOC) content **(A)** and alfalfa aboveground dry-matter yield **(B)**, and their relationships with the DNDC simulated SOC **(C)** and yield **(D)** at six experimental sites across northern China from 2012 to 2017. The solid black line in C and D represents the linear trend fitted to the simulated versus the observed data. ***p < 0.001.

The observed SOC contents (0-30 cm) and alfalfa dry-matter yields at the six experimental sites from 2012 to 2017 are presented in **Figure 5**. The results showed that the SOC varied from 10.17 to 24.48 g kg^-1^ across sites and years. The average SOC was evaluated as 15.23 g kg^-1^. The alfalfa dry-matter yields were measured as 6.49 Mg ha^-1^ on average, with a minimum-maximum range between 4.46 and 9.34 Mg ha^-1^. Alfalfa yields showed an inverse trend against the latitudes. Yields from sites in the higher latitudes (e.g., Hulunber) compared lower than sites in the lower latitudes (e.g., Jiuquan), exposing the climatic influence on alfalfa yield. The DNDC-simulated SOC and alfalfa yield compared well to the observed values on a per-site basis (**Figures 5C, D**). More specifically, over 90% of the observed variations in both SOC and alfalfa yield were captured and reproduced by the DNDC model. The DNDC’s overall prediction accuracy was evaluated at 92.4% for alfalfa yield and 90.7% for SOC.

### 3.2 Predicted future alfalfa yield and SOC contents

**Figure 6:**
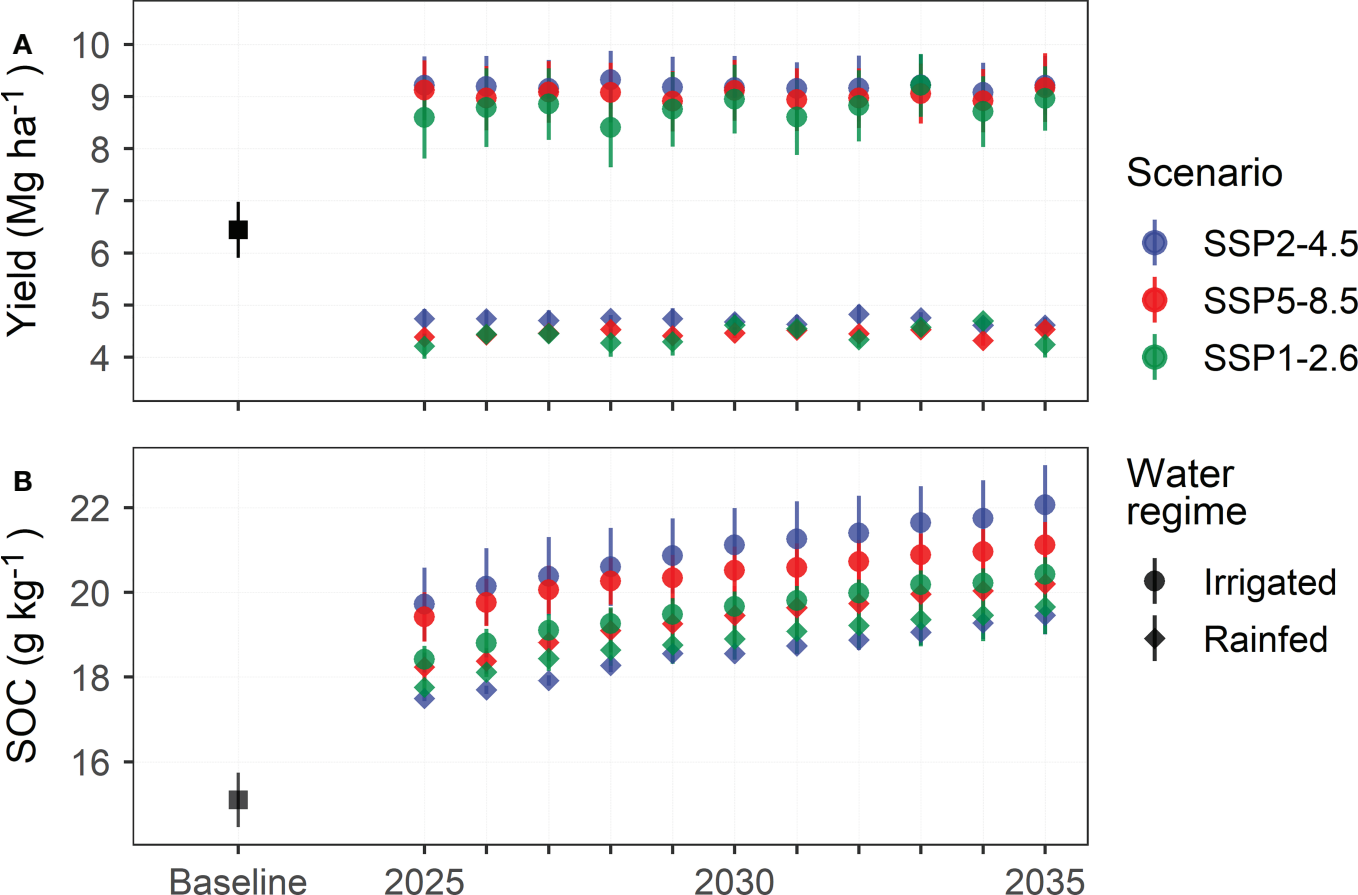
DNDC-simulated average yield **(A)** of and 0-30 cm soil organic carbon content (SOC, **B**) under sown alfalfa pastures on marginal land in northern China under three IPCC SSP scenarios from 2025 to 2035. Experimental observations from 2012 to 2017 are included as the baseline for comparison.

The dry-matter yield of sown alfalfa pastures on marginal lands from 2025 to 2035 in northern China was projected by the DNDC model to fluctuate slightly around a level of 4.5 Mg ha^-1^ in rainfed and 9.0 Mg ha^-1^ in irrigated conditions (**Figure 6**). The rainfed yield compared lower than the site-observed average yield of 6.6 Mg ha^-1^, while the irrigated yield compared substantially higher, reflecting the fact that the site experiments of alfalfa trials involved only partial irrigation to avoid plant failure because of drought. A slightly decreasing tendency in alfalfa yield during 2025-2035 was captured by the DNDC model, confirming the yield pattern of Chinese cultivars as also revealed by experiments on the Loess Plateau (Jiang et al., 2006). Moreover, the projected alfalfa yield tended to be the highest with the SSP2-4.5 climate scenario, followed by SSP5-8.5 and SSP1-2.6. The average irrigated yield was projected as 9.19, 9.03, and 8.79 Mg ha^-1^, whereas the average rainfed yield was projected as 4.71, 4.46, and 4.43 Mg ha^-1^ under the three SSP scenarios, respectively, showing that the SSP-induced yield differences were much smaller than irrigation-induced differences. The DNDC model projected temporally consistent patterns for irrigated yield across the three SSP scenarios. However, the same pattern was hardly discernable for rainfed yield, especially between the SSP5-8.5 and SSP1-2.6 scenarios.

The SOC content in the marginal soil under sown alfalfa pastures in northern China was projected to increase at a consistently positive rate of 0.19 g kg^-1^ yr^-1^ across water regimes and climate scenarios between 2025 and 2035. The projected SOC levels under rainfed and irrigated conditions were averaged at 18.9 and 20.3 g kg^-1^, respectively, corresponding to a net increase in SOC by 8.47 and 9.88 g kg^-1^ above the fallow management. Moreover, the projected SOC increase was compared considerably lower than the site-observed SOC increase of 2.01 g kg^-1^ yr^-1^, showing that DNDC well represented the differences between plot trials and field production. The projected SOC content also displayed a clear high-low differentiation across the climate scenarios and water regimes from 2025 to 2035. The SOC content under irrigated alfalfa pastures tended to be the highest with the SSP2-4.5 scenario (14.00 g kg^-1^), followed by SSP5-8.5 (13.61 g kg^-1^) and SSP1-2.6 (13.05 g kg^-1^) scenarios. It is worth noting that the SOC content under rainfed alfalfa pastures was the lowest with the SSP2-4.5 climate scenario (12.36 g kg^-1^), compared to 12.57 g kg^-1^ with SSP1-2.6 and 12.90 g kg^-1^ with SSP5-8.5 scenarios, showing that the climate sensitivity of SOC content was well reproduced by the DNDC simulation results.

### 3.3 Impact on grazing intensity

**Figure 7:**
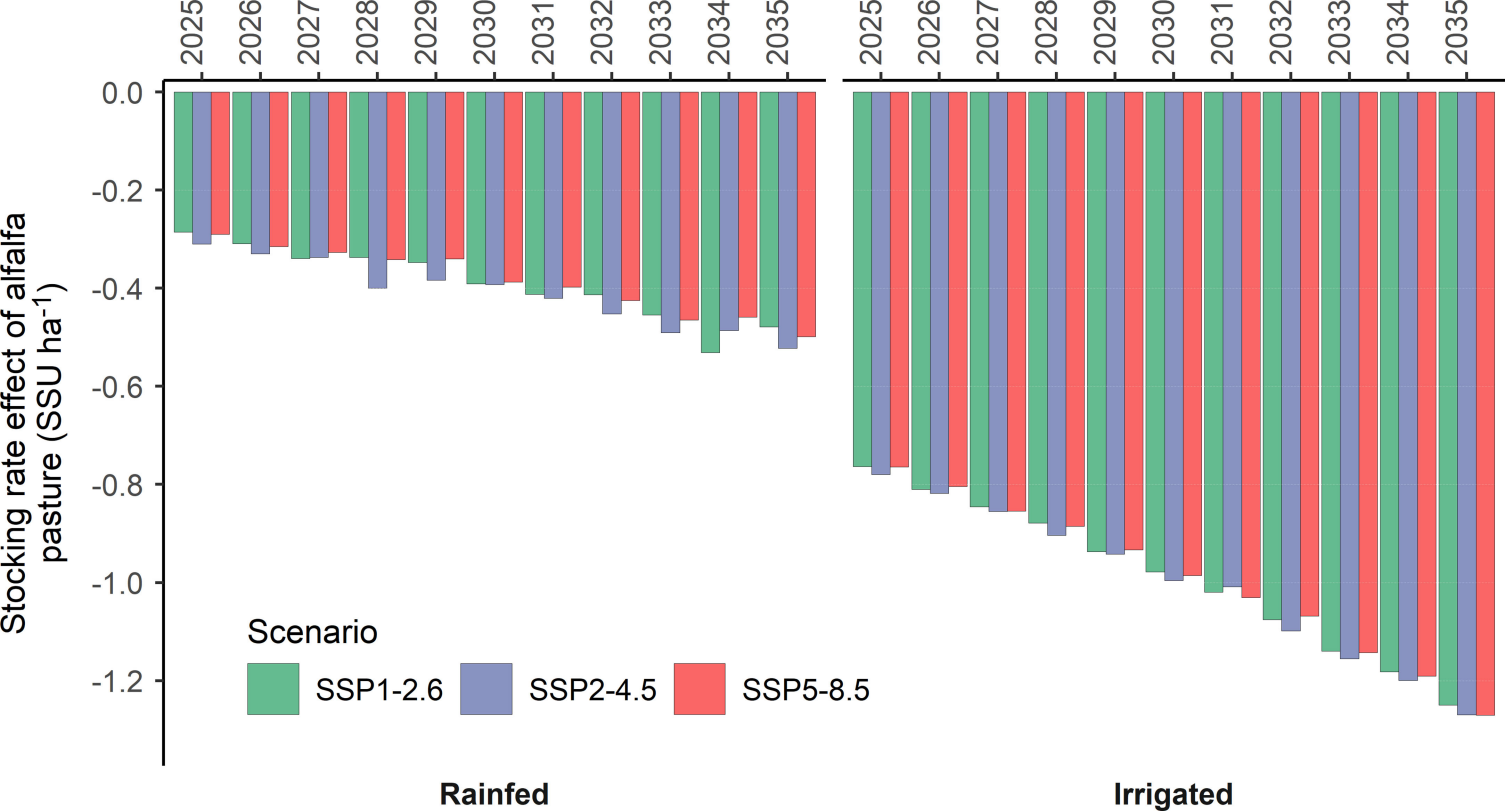
The net change in natural grassland’s stocking rate in response to sown alfalfa pasture on marginal lands in northern China under three IPCC SSP scenarios. SSU, standard sheep unit.

If half of the available marginal lands in northern China were utilized, the forage production from sown alfalfa pastures would increase from 0.83 million Mg in 2025 to 1.42 million Mg in 2035 under rainfed and from 2.08 million Mg in 2025 to 3.42 million Mg in 2035 under irrigated conditions (**Table S2**). The alfalfa forage production variated only slightly across the SSP climate scenarios. The average rainfed alfalfa forage production was projected to be 1.11, 1.16, and 1.09 million Mg under the SSP1-2.6, SSP2-4.5, and SSP5-8.5 scenarios, respectively, whereas the irrigated forage production was projected to be 2.69, 2.72, and 2.68 million Mg under the same scenarios. This corresponds to an increase in the livestock supporting capacity from 17.26 million stand sheep units (SSU, derived from livestock numbers using a conversion factor of 1 for sheep, 0.8 for goats, and 5 for cattle, horses, and camels (NBSC, 2021)) in 2025 to 29.26 million SSU in 2035 under the rainfed conditions. Likewise, the sown alfalfa pastures’ livestock-supporting capacity would increase from 45.07 million SSU in 2025 to 74.09 million SSU in 2035 under irrigated conditions. Effectively, due to the displacement of market demands by livestock production in captivity, the livestock stocking rate on natural grasslands in northern China would decrease by 0.29 and 0.77 SSU ha^-1^ or 7.8% and 20.4% under rainfed and irrigated conditions in 2025. The grassland stocking rate was projected to decrease continuously during the 2025-2035 period. In 2035, the stocking rate would decrease by 0.50 SSU ha^-1^ or 13.2% under rainfed and 1.26 SSU ha^-1^ or 33.5% under irrigated conditions (**Figure 7**). Full details on alfalfa pasture acreage and stocking rate evaluations are given in **Table S2** of the **Supplementary Material**.

### 3.4 Impact on SOC density

**Figure 8:**
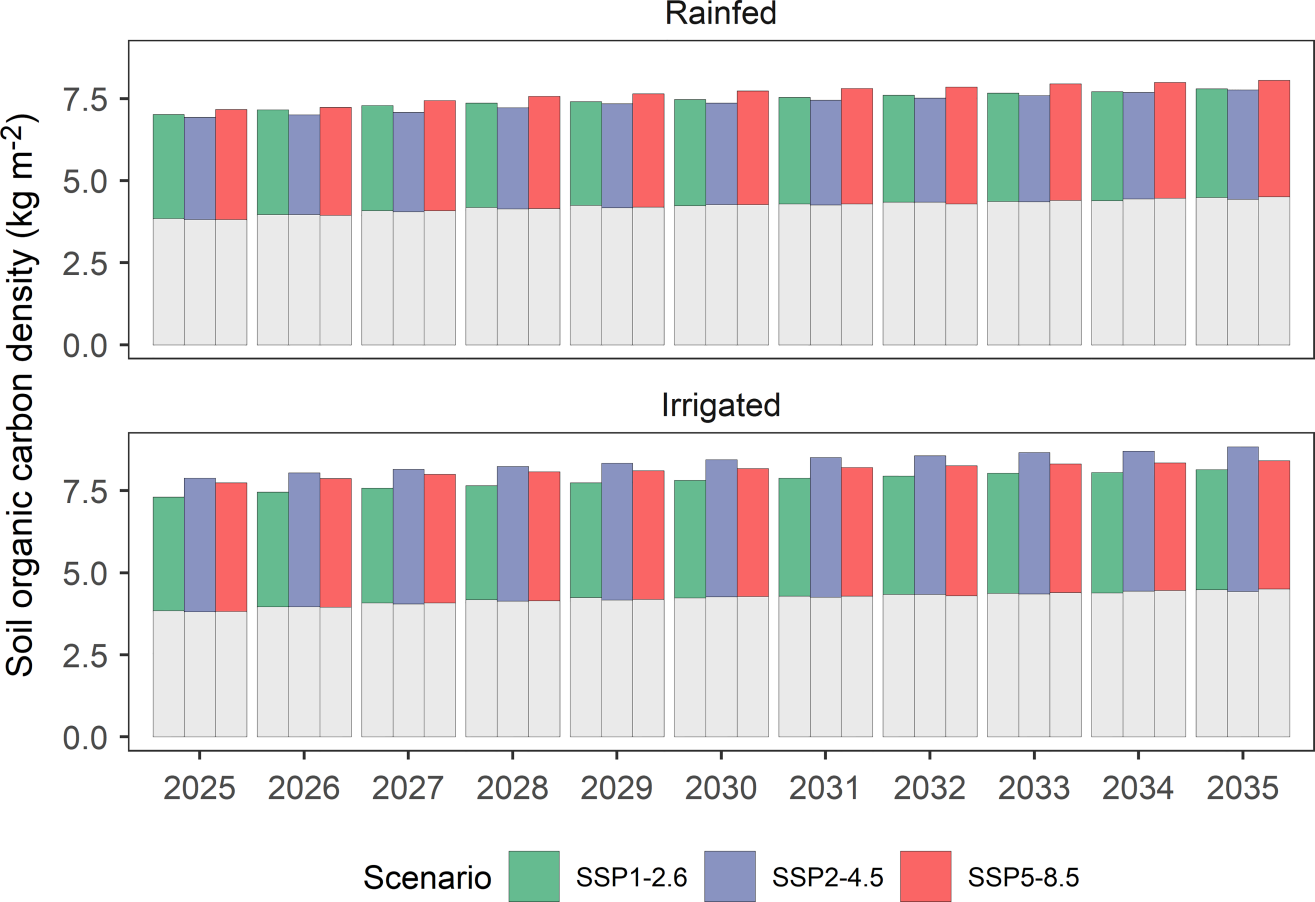
Variations in marginal land soil organic carbon density as affected by sown alfalfa pastures in northern China under three IPCC SSP scenarios from 2025 to 2035 based on DNDC simulations. Grey bars represent the bare soil, whereas color bars represent the net effect of sown pastures on soil organic carbon density.

The SOC density (SOCD) in marginal soils under sown alfalfa pasture in northern China was projected to increase significantly at an average rate of 0.08 kg m^-2^ year^-1^, from 7.34 kg m^-2^ in 2025 to 8.17 kg m^-2^ in 2035 (**Figure 8**). It is worth noting that the DNDC model also projected positive effects of fallow management on SOCD. The SOCD of fallowed marginal lands in northern China was projected to increase from 3.83 kg m^-2^ in 2025 to 4.48 kg m^-2^ in 2035. Taken together, the sown alfalfa pasture on marginal lands in northern China may cause a net increase in SOCD at magnitudes ranging between 3.51 kg m^-2^ and 3.69 kg m^-2^ during 2025-2035. The projected SOCD varied slightly among the SSP climate scenarios. The highest SOCD level (7.68 kg m^-2^) was found in rainfed alfalfa pasture under the SSP5-8.5 scenario, whereas in irrigated alfalfa pasture, the highest SOCD (8.40 kg m^-2^) was found under the SSP2-4.5 scenario with an overall standard deviation of 0.82 kg m^-2^.

## 4 Discussion

### 4.1 Model prediction accuracy

Previous research (Li et al., 2016; Khalil et al., 2020) found that pasture systems were less involved in DNDC model development and, consequently, the number of model applications to pasture systems was much less than to, e.g., cropland systems. To overcome the hurdle of insufficient DNDC modeling expertise for alfalfa pastures in northern China per se, we adopted a field experiment-based calibration approach in modeling alfalfa pasture’s forage production and C sequestration potentials. We collected daily data on soil moisture, soil temperature, and soil greenhouse gas flux for at least two alfalfa growing seasons to calibrate the DNDC model. We also collected yield and SOC data from six experimental sites between 2012 and 2017. These efforts were well rewarded by the obtained accuracy of the modeling results. We obtained an overall prediction accuracy of 90.73% for SOC and 92.41% for alfalfa’s dry-matter yield, aggregated from the six experimental sites across northern China. We also obtained high goodness-of-fit levels between the model-predicted and field-observed SOC and yield. The coefficient of determination (R^2^) was evaluated as 0.85 for SOC and 0.88 for yield. In general, our modeling results achieved higher accuracy than most of the DNDC model applications in China and elsewhere during the past five years (**Table 4**). Moreover, the model reproduction of the observed variability patterns in space and time for both SOC and yield was well done, as shown by the specific scatter plots (**Figures 4B, D, F**; **Figures 5C, D**), which were rarely provided in previous DNDC model applications, in addition to time-series plots (**Figures 4A, C, E**, **5A, B**).

**Table 4:** Comparison of the DNDC model’s yield and soil organic carbon (SOC) prediction accuracies between this study and other studies found in the recent literature.

Source	Index	Location	RMSE	MAE	MAPE	R^2^
*This study*	SOC	China	0.17 g kg^-1^	0.15 g kg^-1^	9.27%	0.85
Han et al. (2017)	SOC	China	3.80 g kg^-1^	2.28 g kg^-1^	15.27%	0.69
Zhang et al. (2017)	SOC	China	0.92 g kg^-1^	0.74 g kg^-1^	9.89%	0.50
Li et al. (2016)	SOCD	China	0.24 kg m^-2^	0.17 kg m^-2^	6.92%	0.73
Liao et al. (2016)	SOCD	China	0.33 kg m^-2^	0.26 kg m^-2^	12.03%	0.30
Singh and Benbi (2020)	SOCD	India	0.09 kg m^-2^	0.08 kg m^-2^	11.45%	0.78
Khalil et al. (2020)	SOCD	UK	1.07 kg m^-2^	0.93 kg m^-2^	15.89%	0.49
*This study*	yield	China	0.65 Mg ha^-1^	0.53 Mg ha^-1^	7.59%	0.88
Jiang et al. (2021)	yield	China	0.14 Mg ha^-1^	0.12 Mg ha^-1^	1.21%	0.94
Li et al. (2017)	yield	China	1.22 Mg ha^-1^	0.87 Mg ha^-1^	12.11%	0.45
Pu et al. (2019)	yield	China	1.53 Mg ha^-1^	1.25 Mg ha^-1^	20.05%	0.73
Zhang et al. (2017)	yield	China	1.05 Mg ha^-1^	0.79 Mg ha^-1^	27.34%	0.74
He et al. (2021)	yield	Canada	1.13 Mg ha^-1^	0.65 Mg ha^-1^	23.18%	0.76
He et al. (2019)	yield	Canada	1.16 Mg ha^-1^	0.85 Mg ha^-1^	15.26%	0.87
Wang et al. (2021)	yield	Canada	1.66 Mg ha^-1^	1.09 Mg ha^-1^	18.76%	0.78
Jarecki et al. (2018)	yield	Canada	0.39 Mg ha^-1^	0.35 Mg ha^-1^	4.55%	0.95

SOCD, soil organic carbon density.

In comparison, Han et al. (2017), for example, simulated cropland SOC in China with the DNDC model and obtained a satisfactory prediction accuracy of 84.73% using country-wide soil and crop management data, which corresponded to a mean absolute percentage error (MAPE) of 15.27%. Likewise, Zhang et al. (2017) modeled the 0-20 cm SOC of the wheat-maize system in the North China Plain based on a 30-year experimental record and reached a prediction accuracy of 90.11%. Moreover, Khalil et al. (2020) simulated the SOC density in the 0-15 cm soil layer of a ryegrass pasture in the U.K. using the DNDC model and managed to keep the prediction error lower than 16%, a level comparable to Han et al. (2017). However, the coefficient of determination (R^2^) for Khalil et al.’s simulation results (0.49) was substantially lower than that of Han et al. (0.69), showing that DNDC is less capable of accounting for field variabilities in pasture soil C than in cropland systems. This accountability discrepancy is in part due to the low quality of input data, as elaborated by Li et al. (2016). In the wheat-maize system studied by Li et al. (2016), straw retention was the major C input into the soil. A rough estimation of the straw retention rate at the regional scale would naturally lead to low accountability of spatial variability in the modeling results. This was in sharp contrast to this study where all calibration data were experimentally collected at the plot scale.

Much attention has been given to yield prediction in almost all modeling efforts including DNDC. Although yield prediction was not the primary focus of the DNDC model (Li et al., 1992), the DNDC model was more and more frequently applied in yield prediction in recent years. For example, Jiang et al. (2021) simulated the yield of paddy rice in China and obtained a high prediction accuracy of 98.79%. Jarecki et al. (2018) simulated the yield of the maize-alfalfa system in Canada, which achieved an accuracy of 95.45%. Although the yield prediction accuracy of this study was slightly lower than these two examples, the yield prediction accuracy of this study was higher than all the rest studies listed in **Table 4**. It is worth noting that the high prediction accuracy of this study was achieved for a pasture system that was not as well supported by the DNDC model as for a cropland system. Moreover, the high prediction accuracy of this study was achieved for northern China, where DNDC modeling expertise was much poorer than, e.g., in Canada (He et al., 2019).

### 4.2 Effects on grazing intensity

Many studies (Zhang et al., 2015; Gao et al., 2016; Kemp et al., 2020) have suggested that overgrazing was one of the most important drivers of grassland degradation in China. A recent survey found that nearly 90% of the grasslands in northern China experienced varying degrees of degradation (Kemp et al., 2020) and these degraded grasslands were overgrazed by 27-89% (Zhang et al., 2014). Previous research suggested that grazing intensity control should be used as the primary means of grassland conservation (Zhang et al., 2015). In most areas in northern China, a 50% reduction in grazing intensity was advocated. However, it remained uncertain how these grazing intensity reduction targets can be realized in practice, especially at the regional scale. Although livestock rearing in captivity has been proposed as a pragmatic measure to displace market pressures away from the grasslands (Chen et al., 2017), it relies on imported forage which has complex implications for the domestic food market. In this study, we demonstrated through DNDC model simulation that sown alfalfa pasture on marginal land has the potential to effectively lower the grazing intensity in northern China by, on average, 8-13% under climate change in the near-term future. We also demonstrated that the grazing intensity on natural grasslands in northern China could be reduced by one-third (20-33%) if irrigated alfalfa pasture had been regionally implemented. This represents significant progress in grassland protection due to multiple reasons.

Firstly, sown alfalfa pasture on marginal land represents a practical solution based on the integrated use of land resources. Since the beginning of the 21^st^ century, a range of policy reforms on the ecological protection of grassland resources has been instituted in China. These included the Grassland Eco-compensation Program, the Farmer’s Professional Cooperative Law, and so on, in addition to the well-known Natural Grassland Restoration Program and the Returning Grazing Land to Grassland Program (Robinson et al., 2017). Collectively under these institutions, marginal lands, which were previously under agricultural cultivation, were set aside on fallow. In 2020, there were 4,000 km^-2^ of marginal land under fallow management or being regarded as a wasteland in northern China (NBSC, 2021). In this study, we considered a 50% use of these marginal land resources for sown alfalfa pasture in 2025. From 2026 to 2035, an annual expansion in sown alfalfa pasture areas of 5% was considered (**Table S2**).

Secondly, sown alfalfa pasture represents the single most effective technical measure at the regional scale which has the potential to, if implemented on 100% of the marginal land area, lower the grazing intensity on northern China grasslands by at least 25 (rainfed) to 66% (irrigated) in changing climate conditions. Effectively, this will eliminate overgrazing on about half of the degraded grasslands in northern China, based on a comparison to the evaluation results of Zhang et al. (2014).

Thirdly, sown alfalfa pasture in combination with marginal lands in northern China also has social significance for herder households. A lower grazing intensity does not necessarily mean lower household income, because productivity per head of animal usually increases with decreasing herd size (Zhang et al., 2015). More importantly, a decreased herd size will hopefully spare time for education and training (Gao et al., 2016), which is much needed for herder households across the region.

### 4.3 Effects on soil C sequestration

Grasslands store approximately one-third of the global terrestrial C. On the one hand, improvements in grazing management and biodiversity restoration have the potential to deliver natural climate benefits in grasslands through soil C sequestration (Yao et al., 2015; Li et al., 2022). On the other hand, however, widespread grassland degradation poses significant threats to the soil C pool by enhanced soil C mineralization (Ye et al., 2010; Abdalla et al., 2018). The critical role of soil C in global climate change regulation has not only been recognized by scientists but also by policymakers. Notably, at the 21st session of the United Nations Framework Convention on Climate Change, which was held in Paris in 2015, an ambitious target of a 0.4% rate of the annual increase in global SOC stock was proposed under the Agenda for Actions (Soussana et al., 2019). This target of a 0.4% increase in global SOC stocks, codenamed “4 per Mille”, was evaluated as the amount of C sequestration required per year to offset anthropogenic greenhouse gases emissions of 8.9 Giga tons (Minasny et al., 2017). In this study, significant SOC increases were observed in experimental plots under sown alfalfa pastures at all 6 sites across northern China. In 2012, the site averaged SOC was measured at 11.4 g kg^-1^ when alfalfa seeds were sown. SOC increased to 12.1 g kg^-1^ in 2013, which corresponded to a 5.9% increase. The annual rate of SOC increase was measured at 9.8% in 2014, 10.6% in 2015, 15.8% in 2016, and 31.2% in 2017, which were all more than 10 times higher than the 0.4% threshold, showing alfalfa pasture’s significantly positive effects on soil C sequestration.

**Figure 9:**
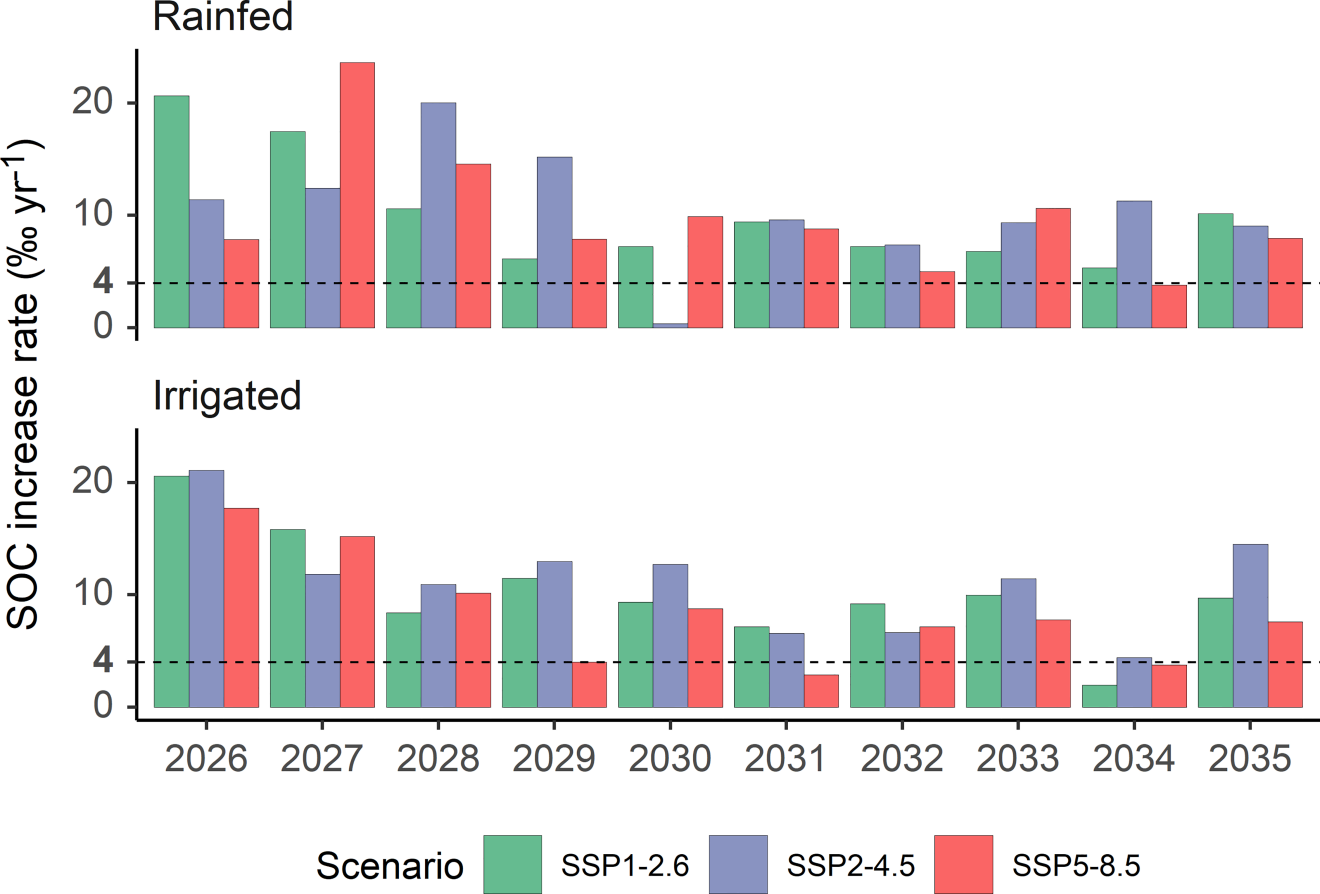
DNDC-simulated increase rate (‰ yr^-1^) of soil organic carbon of marginal land with sown alfalfa pastures in northern China from 2026 to 2035 under three IPCC SSP scenarios. The dashed horizontal line represents the increase rate advocated by the “4 per Mille” initiative (Minasny et al., 2017).

Alfalfa pasture’s positive effects on SOC were not only observed at the site scale but also confirmed by the DNDC model simulations at the regional scale. The marginal land SOC under sown alfalfa pastures in northern China was projected to increase at the rate of 1.31% in 2026, 1.81% in 2027, and 0.93% in 2035, the last year of the simulation period, under the rainfed conditions. All these rates are significantly higher than the 0.4% threshold stipulated by the 4 per Mille Initiative. Under the irrigated conditions, even higher SOC increase rates were projected (**Figure 9**), reflecting the primary influence of C input quantities – both from the aboveground biomass through the litterfall pathway and from the belowground biomass through the root pathway – on soil C sequestration (Chen et al., 2021; Li et al., 2022; Xu et al., 2022).

We would like to indicate that, in addition to C sequestration in marginal lands, sown alfalfa pastures also result in invaluable ecosystem gains via, e.g., grazing intensity control. Previous research found that increasing gazing intensity on global grasslands was associated with decreasing trends of SOC and increasing trends of soil bulk density (Lai and Kumar, 2020). This signifies that lessening grazing intensity is itself an efficient grassland conservation measure that may produce significant indirect benefits for soil fertility conservation and soil water retention. We must also indicate that the so-called restoration of grassland productivity in northern China that was suggested by recent satellite data (Chen et al., 2017) and hailed as a proven achievement of past grassland conservation programs was both superficial and early mature. What the satellite data showed was the growth of aboveground biomass. The more endurable restoration, such as a replenished SOC stock, was hidden from satellite observations (Ma et al., 2017) and is yet to accomplish.

### 4.4 Uncertainties

**Figure 10:**
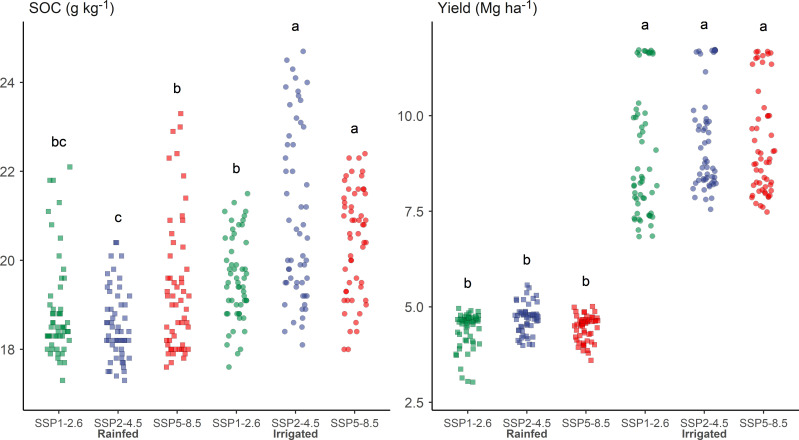
Soil organic carbon (SOC) content and alfalfa forage yield under different climate futures and irrigation assumptions in northern China from 2025 to 2035. Different letters indicate significant difference (p< 0.05).

Uncertainties in future climates represent a major challenge to modeling ecosystem functions (Parker, 2013). One solution to improve the certainty of the modeling outcomes is to adopt an ensemble modeling scheme so that the uncertainty of the modeling outcome can be statistically obtained, as showcased by IPCC-AR6 (IPCC, 2022). In this study, however, we bypassed this heavy-duty option of ensemble modeling but opted to use three contrasting climate scenarios. A high degree of certainty was achieved in the simulated alfalfa yield and SOC, as suggested by the LSD tests applied to these results (**Figure 10**). The LSD tests showed that alfalfa yields were not significantly different across the three SSP scenarios, meaning that alfalfa yield in northern China in the near-term future will be highly likely within the simulated yield range. Although a seemingly more complex picture was depicted by the simulation results for SOC under sown alfalfa pastures, the message that the results conveyed was quite clear. Our climate future will be much more likely along a sustainable development path (SSP1-2.6) than pretending unawareness of climate change (SSP5-8.5). There will be many people taking a middle road (SSP2-4.5), but the difference between this road and SSP1-2.6 was not statistically significant. This means that the marginal land’s SOC content under alfalfa pasture in northern China from 2025 to 2035 will be most likely within the predicted SOC range with the SSP1-2.6 and SSP2-4.5 scenarios.

It is well known that irrigation water is scarce in northern China. The situation will likely not turn better from 2025 to 2035 in the five northern provinces considered here (**Figure 2**). The consideration to include an irrigation scenario in this study was to provide an option for areas where water resources were more abundant and hence a tradeoff among competing uses had economic importance, as in the case of Gansu province where irrigated alfalfa was practiced (Jiang et al., 2006). The statistically significant difference between irrigated and rainfed alfalfa forage yield in **Figure 10** confirmed that irrigation will still be an economic incentive in local water management decisions. An additional ecosystem benefit that irrigated alfalfa will produce is that it significantly increases the soil’s carbon sequestration potential by 20%.

## 5 Conclusions

We have demonstrated through field experiments and DNDC modeling that sown alfalfa pasture on marginal lands in northern China has the potential to lower the livestock grazing intensity on regional grasslands by a margin of 25-66%. We have also demonstrated that the marginal land’s SOC under the sown-alfalfa-pasture management is likely to increase at annual rates higher than 4‰ yr^-1^ from 2025 to 2035 under a most likely future climate. These systematic, firsthand experimental and modeling findings highlight the sown alfalfa pasture’s roles in grassland protection and ecosystem provision at regional scales, showcasing an important pathway to sustainable grassland development under climate change over a short- to medium-term time horizon.

## Data availability statement

The original contributions presented in the study are included in the article/**Supplementary Material**. Further inquiries can be directed to the corresponding authors.

## Author contributions

All authors listed have made a substantial, direct, and intellectual contribution to the work and approved it for publication. All authors contributed to the article and approved the submitted version.

## Funding

This work was supported by the National Natural Science Foundation of China (grant number: 41703081), and the China Agriculture Research System (grant number: CARS-34).

## Conflict of interest

The authors declare that the research was conducted in the absence of any commercial or financial relationships that could be construed as a potential conflict of interest.

## Publisher’s note

All claims expressed in this article are solely those of the authors and do not necessarily represent those of their affiliated organizations, or those of the publisher, the editors and the reviewers. Any product that may be evaluated in this article, or claim that may be made by its manufacturer, is not guaranteed or endorsed by the publisher.
