# Supplementary material for: Sown alfalfa pasture decreases grazing intensity while increasing soil carbon: Experimental observations and DNDC model predictions
Source: Front Plant Sci. 2022 Nov 21;13:1019966. doi: 10.3389/fpls.2022.1019966 (PMC9720138; doi:10.3389/fpls.2022.1019966)
Supplement: Supplementary file 1 [file Table_1.docx]

Supplementary Material

**Table S1.** Adjustment of major DNDC input parameters for six experimental sites in northern China.

| Parameter | Site | Default value | Adjusted value |
| --- | --- | --- | --- |
| Maximal biomass (kg C ha^-1^ year^-1^) | Hulunber | 5,614 | 5,750 |
|  | Suihua | 5,614 | 6,250 |
|  | Chifeng | 5,614 | 6,000 |
|  | Gongzhuling | 5,614 | 5,500 |
|  | Yulin | 5,614 | 6,200 |
|  | Jiuquan | 5,614 | 6,000 |
| Biomass partitioning fraction (grain - shoot - leaf- root) | Hulunber | 0.50 - 0.05 - 0.05 - 0.40 | 0.10 - 0.30 - 0.15 - 0.45 |
|  | Suihua | 0.50 - 0.05 - 0.05 - 0.40 | 0.08 - 0.25 - 0.30 - 0.37 |
|  | Chifeng | 0.50 - 0.05 - 0.05 - 0.40 | 0.10 - 0.25 - 0.30 - 0.35 |
|  | Gongzhuling | 0.50 - 0.05 - 0.05 - 0.40 | 0.10 - 0.20 - 0.30 - 0.40 |
|  | Yulin | 0.50 - 0.05 - 0.05 - 0.40 | 0.10 - 0.30 - 0.30 - 0.30 |
|  | Jiuquan | 0.50 - 0.05 - 0.05 - 0.40 | 0.15 - 0.30 - 0.30 - 0.25 |

(Continued on page 2)

| Parameter | Site | Default value | Adjusted value |
| --- | --- | --- | --- |
| Biomass C/N (grain - shoot - leaf- root) | Hulunber | 10 - 10 - 10 - 35 | 20 - 10 - 10 - 13 |
|  | Suihua | 10 - 10 - 10 - 35 | 20 - 10 - 10 - 13 |
|  | Chifeng | 10 - 10 - 10 - 35 | 20 - 10 - 10 - 13 |
|  | Gongzhuling | 10 - 10 - 10 - 35 | 20 - 10 - 10 - 13 |
|  | Yulin | 10 - 10 - 10 - 35 | 20 - 10 - 10 - 13 |
|  | Jiuquan | 10 - 10 - 10 - 35 | 20 - 10 - 10 - 13 |
| Accumulative thermal unit (growing degree-days) | Hulunber | 2,000 | 2,500 |
|  | Suihua | 2,000 | 2,200 |
|  | Chifeng | 2,000 | 2,200 |
|  | Gongzhuling | 2,000 | 2,200 |
|  | Yulin | 2,000 | 2,050 |
|  | Jiuquan | 2,000 | 1,950 |
| Water demand ratio (g water/g dry matter) | Hulunber | 300 | 100 |
|  | Suihua | 300 | 200 |
|  | Chifeng | 300 | 200 |
|  | Gongzhuling | 300 | 250 |
|  | Yulin | 300 | 250 |
|  | Jiuquan | 300 | 250 |

(Continued on page 3)

| Parameter | Site | Default value | Adjusted value |
| --- | --- | --- | --- |
| Nitrogen fixation ratio (total plant N/plant N taken from soil) | Hulunber | 4 | 3 |
|  | Suihua | 4 | 3 |
|  | Chifeng | 4 | 3 |
|  | Gongzhuling | 4 | 3 |
|  | Yulin | 4 | 3 |
|  | Jiuquan | 4 | 3 |
| Optimal temperature (°C) | Hulunber | 23 | 15 |
|  | Suihua | 23 | 20 |
|  | Chifeng | 23 | 20 |
|  | Gongzhuling | 23 | 15 |
|  | Yulin | 23 | 15 |
|  | Jiuquan | 23 | 15 |
